# Modeling and Prediction of the Species’ Range of *Neurobasis chinensis* (Linnaeus, 1758) under Climate Change

**DOI:** 10.3390/biology11060868

**Published:** 2022-06-06

**Authors:** Jian Liao, Haojie Wang, Shaojun Xiao, Zhaoying Guan, Haomiao Zhang, Henri J. Dumont, Bo-Ping Han

**Affiliations:** 1 Department of Ecology, Institute of Hydrobiology, Jinan University, Guangzhou 510632, China; liaojian@stu2017.jnu.edu.cn (J.L.); whjie@stu2020.jnu.edu.cn (H.W.); henri.dumont@ugent.be (H.J.D.); 2 Guangdong Lianshan Bijiashan Provincial Nature Reserve Administration Bureau, Qingyuan 513200, China; xiaoshaojun790616@163.com; 3 School of Applied Biology, Shenzhen Institute of Technology, Shenzhen 518055, China; guanzhaoying717@126.com; 4 State Key Laboratory of Genetic Resources and Evolution, Kunming Institute of Zoology, Chinese Academy of Sciences, Kunming 650201, China; zhanghaomiao@mail.kiz.ac.cn; 5 Department of Biology, Ghent University, 9000 Ghent, Belgium

**Keywords:** *Neurobasis chinensis*, climate change, MaxEnt model, potential distribution, core habitat

## Abstract

**Simple Summary:**

Global climate change is accelerating and modifying the distribution of many extant species. Dragonflies, as a group, inhabit aquatic as well as terrestrial environments and are considered sensitive climate change indicators. In this study, we model and predict the range of a large, tropical damselfly *Neurobasis chinensis* L. under the last glacial maximum (LGM), the current, and four future warming scenarios. The models show that the species mainly occupies forest ecosystems below 1200 m (preferring 500 to 1200 m) and had two historic core distribution areas in LGM, one of which survived, namely south-central Vietnam. The future scenarios show that the core distribution, high suitable habitats, and even the whole species range of *N. chinensis* will extend northwards.

**Abstract:**

*Neurobasis chinensis* is widely distributed in eastern tropical Asia. Its only congener in China, the *N. anderssoni*, has not been observed for decades. To protect *N. chinensis*, it is necessary to understand the ecological properties of its habitats and specie’s range shift under climate change. In the present study, we modeled its potential distribution under one historical, current, and four future scenarios. We evaluated the importance of the factors that shape its distribution and habitats and predicted the historical and current core spatial distributions and their shifting in the future. Two historical core distribution areas were identified: the inland region of the Bay of Bengal and south-central Vietnam. The current potential distribution includes south China, Vietnam, Laos, Thailand, Myanmar, Luzon of Philippines, Malaysia, southwest and northeast India, Sri Lanka, Indonesia (Java, Sumatera), Bangladesh, Nepal, Bhutan, and foothills of the Himalayas, in total, ca. 3.59 × 10^6^ km^2^. Only one core distribution remained, concentrated in south-central Vietnam. In a warming future, the core distribution, high suitable habitats, and even the whole range of *N. chinensis* will expand and shift northwards. Currently, *N. chinensis* mainly resides in forest ecosystems below 1200 m above sea level (preferred 500 m to 1200 m a.s.l.). Annual precipitation, mean temperature of driest quarter, and seasonality of precipitation are important factors shaping the species distribution. Our study provides systematic information on habitats and geographical distribution, which is useful for the conservation of *N. chinensis*.

## 1. Introduction

The large damselfly *Neurobasis chinensis*, conspicuous by its iridescent metallic colors in the male, was the first dragonfly recorded from China in the 10th edition of Linnaeus’s Systema Naturae of 1758 [1]. It is the most widespread species in the genus *Neurobasis* [2]. The genus of *Neurobasis* contains 13 known species, which are all distributed in the eastern tropics of Asia [1,2]. With the exception of *N. chinensis*, other *Neurobasis* species are rare or locally endemic (many are island species), and some are endangered or extinct [1,2]. *Neurobasis kaupi*, for example, is restricted to the island of Sulawesi [2]. Such species have a high extinction risk under habitat loss. IUCN red list category and criteria assessed *Neurobasis daviesi* as the level of Vulnerable D2, which is endangered (https://www.iucnredlist.org/species/139120960/146601168, Version 2021–3, accessed on 18 April 2022). *Neurobasis anderssoni*, the only congener of *N. chinensis* in China, has not been observed for decades and is considered extinct [1]. In order to promote future conservation efforts for extant species, we need to recognize the properties of their habitats, core spatial distribution, and factors disturbing and shaping the species’ range. The range of *N. chinensis* is limited to the eastern tropics of Asia (East Asia, South Asia, Southeast Asia), including China, Vietnam, Laos, Myanmar, Thailand, India, Sri Lanka, Bangladesh, Nepal, Peninsular Malaysia, and Sumatra in South [3,4]. Comparatively, *N. anderssoni* is endemic to China, recorded from Sichuan, Zhejiang, Fujian, and Guangxi, and its range is smaller and more northern than that of *N. chinensis*. *N. chinensis* prefers small, shallow, partially shaded rivers in forests. It usually perches on exposed rocks and oviposits in stream vegetation with a preference for grassy river borders. It also breeds in some disturbed habitats [5]. It frequently appears in hill streams within a span of 2500 m a.s.l. (the highest occurrence recorded is in the Nilgiri Hills, India) above sea level, but it most commonly occurs between 500 and 2000 m a.s.l. [2,4].

As climate variables are critical to the life history of *N. chinensis* [4], it tends to colonize new habitats and shift its distribution under global warming. Global climate change is becoming more and more visible, and, consequently, geographic areas corresponding to ecological zones are changing and affecting the distribution of many plants and animals [6,7]. The 5th Assessment Report (AR5) by the Intergovernmental Panel on Climate Change (IPCC) stated that global warming is expected to continue with the average temperature of Earth increasing by 0.3–4.5 °C by 2100 compared with the 1986–2005 period [8,9]. Unlike the dramatic effects of extremely high or low temperature on insect physiology, the large-scale, long-term, and relatively mild environmental temperature change will result in insect distributions shifting to higher altitudes and latitudes [10,11]. For example, due to the increase in average temperature, forest insects such as *Operophtera brumata* (winter moth), *Epirrita autumnata* (autumnal moth), and *Dendroctonus ponderosae* (mountain pine beetle) extended their areas to higher latitudes and to the north [10,11,12,13].

Species distribution, habitat selection, and dispersal are mainly influenced by meteorological elements [14,15,16]. Climate change is modifying species distribution on a large scale [14,17]. The maximum entropy model (MaxEnt) provides an efficient way to model and predict species distribution with species occurrence records and environmental variables [14,18]. The model has the advantages of high prediction accuracy, simple and fast operation, and small sample requirements [19]. It has been applied in conservation biology [20,21], invasion biology [22], and genetic geography [23]. The present study aims to explore the potential distribution of *N. chinensis* under historical and current climates and its possible shift in the future and to investigate the important variables shaping the distribution.

## 2. Materials and Methods

### 2.1. Data Collection

**Figure 1:**
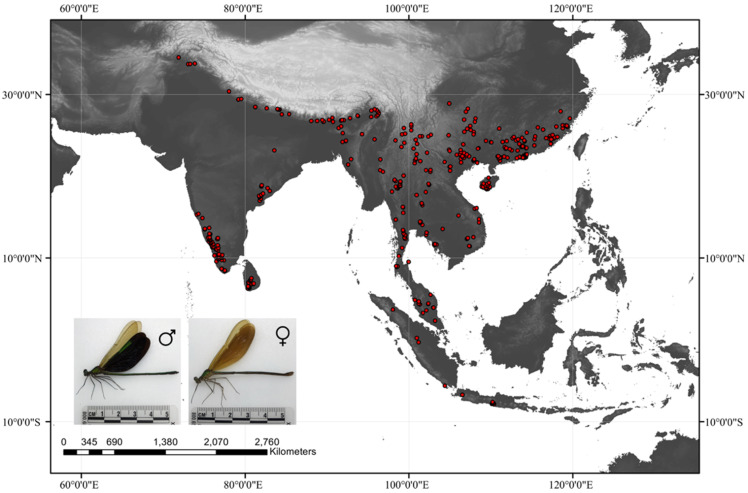
Occurrence records of *N. chinensis* in the world. Data were obtained from literature, the GBIF database, and our fieldwork. The female *N. chinensis* in the bottom left was from Nankunshan Provincial Nature Reserve, Guangdong Province, and the male was from Shiwandashan National Forest Park, Guangxi Province. Pictures were both taken by Jian Liao in 2021.

We obtained the occurrence records of *N. chinensis* across its species range using three methods: extensive literature retrieval, downloading from the Global Biodiversity Information Facility (GBIF, https://www.gbif.org/, accessed on 18 April 2022), and combined with our field investigation works from 2011 to 2012 and from 2016 to 2021. When occurrence records lacked exact geo-coordinates, we obtained the latitude and longitude by using Google Earth (http://ditu.google.cn/, accessed on 18 April 2022). A sighting point map was developed by ArcGIS 10.7 (Esri, Redlands, CA, USA) and then created a 5 km × 5 km grid. The points except those closest to the center of the grid cell are removed so that only one point occurs within each grid cell. Finally, a total of 532 documented presence records of *N. chinensis* (64 sites from literature, 383 from GBIF, and 85 from our previous fieldwork) were obtained for input to the modeling (Figure 1).

### 2.2. Environmental Variables

We initially selected 20 environmental factors that may influence the distribution of *N. chinensis* for the next simulation. These included 19 bioclimatic variables and one elevation parameter (Table 1). All the environment layers, including current (interpolations of observed data, representative of 1970–2000), historical (the last glacial maximum, LGM), and future (the 2100s) layer parameters, were obtained from WORLDCLIM 2.0 at 2.5 min resolution (ca. ~5 × 5 km^2^ at ground level) (https://www.worldclim.org/data/bioclim.html, accessed on 18 April 2022) [24]. In terms of future climate, the four shared socioeconomic pathways (SSPs) represent different levels of carbon emissions: (i) the low-end SSP1-2.6, (ii) the middle SSP2-4.5, (iii) the medium-high SSP3-7.0 and (iv) the worst-case SSP5-8.5 (stabilizes radiative forcing at 2.6 W m^−2^, 4.5 W m^−2^, 7.0 W m^−2^, and 8.5 W m^−2^), approximately 376 ppm, 650 ppm, 1011 ppm and 1228 ppm CO_2_-equivalent in 2100 [25].

To avoid autocorrelated environmental factors, we removed the factors with multicollinearity by using USDM version 1.1-18 in R [26]. We calculated the variance inflation factor (VIF = 1/(1 − Ri^2^)), removed those with a VIF value greater than 10, and 9 environmental factors were selected as evaluator variables: BIO2, BIO5, BIO8, BIO9, BIO12, BIO14, BIO15, BIO18, and BIO19. The Jackknife test was performed based on the current scenario to assess the relative importance of environment variables [14].

### 2.3. Distribution Modeling and Statistical Analysis

MaxEnt model version 3.3.3k [14] (http://www.cs.princeton.edu/, accessed on 18 April 2022) was applied to predict the distribution range areas of *N. chinensis* by using occurrence records and environment variables filtered by autocorrelation analysis (current, historical, and future environments). To estimate the capacity of the model, 25% of the data was used for testing, while 75% was used for training. The algorithm ran 1000 iterations of these processes or continued until they converged (threshold 0.00001). A threshold-independent receiver-operating characteristic (ROC) analysis was carried out to evaluate the performance of MaxEnt and then calculate the area under receiver-operating characteristic curve (AUC) values. AUC value is used to evaluate model accuracy, and there are five conditions: excellent (>0.9), good (0.8~0.9), fair (0.7~0.8) and poor (0.6~0.7) and fail (<0.6). The final potential species distribution map was used to calculate the area of the potential suitable habitats according to the suitability value (range from 0 to 1) in ArcToolbox of ArcMap version 10.7 by 3D Analyst Tool. If the suitability value was closer to 1, the species was to occur more likely in that habitat. All potential habitats were classified into four subdivisions according to the suitability values: ‘high’ (>0.6), ‘moderate’ (0.4–0.6), and ‘low’ potential habitats (0.2–0.4), as well as ‘not potential habitat’ (<0.2) [8]. If the suitability values are greater than 0.8, the habitats were considered a core distribution area. To detect the elevation effect and habitat selection, we extracted and analyzed the elevation layer (from WORLDCLIM 2.0) and ecosystem type layer (from Resource and Environment Science and Datacenter, China, https://www.resdc.cn/data.aspx?DATAID=198, accessed on 18 April 2022), as well as the suitability value of each site in the Spatial Analysis tool in ArcGIS. All the ecosystems in which the habitats are located were classified into disturbed (farmland ecosystem, wetland ecosystem, and settlement ecosystem) and undisturbed habitats (forest ecosystem and grassland ecosystem). Then, nonlinear regression was conducted to demonstrate the elevation effect, and the one-way ANOVA was performed to test the differences among five ecosystem types. All the statistical analyses were performed in SPSS 25 (IBM, Armonk, NY, USA), and the figures were generated with Origin Pro 2022 (OriginLab, Northampton, MA, USA).

## 3. Results

### 3.1. Prediction of Current Suitable Habitat Areas and Model Accuracy

**Figure 2:**
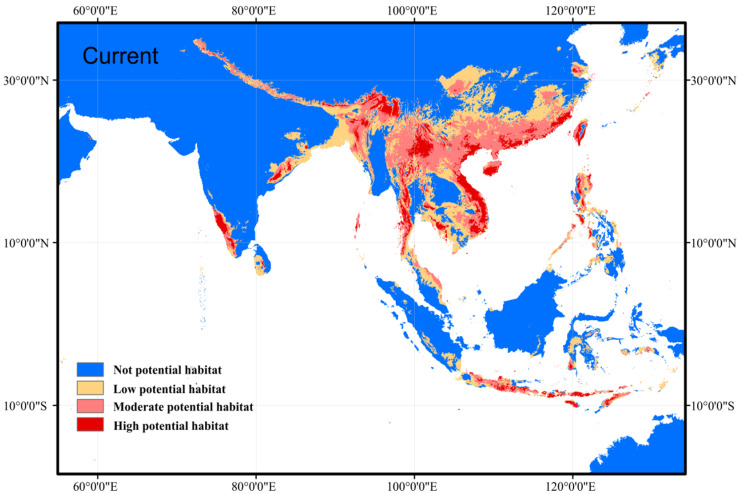
Current distribution area with different potential habitats of *N. chinensis* according to occurrence records. Except for blue areas, all colors represent potential habitat distribution. Potential habitats were classified into high potential habitats (>0.6), moderate potential habitats (0.4~0.6), and low potential habitats (0.2~0.4) according to suitability value.

The average AUC values were 0.929 and 0.914 for the training data set and test data set, respectively, indicating that the MaxEnt model can accurately predict the locations of potential suitable habitats. The current potential distribution map was drawn based on suitability, including high potential habitats, moderate potential habitats, and low potential habitats (Figure 2). The identified potential suitable habitats of *N. chinensis* were located in south China (including the Hainan Island and Taiwan Island), Vietnam, Laos, Thailand, Myanmar, Luzon of Philippines, Malaysia, southwest and northeast India, Sri Lanka, Indonesia (Java, Sumatera), Bangladesh, Nepal, Bhutan and foothills of the Himalayas (Figure 2), in total, covered ca. 3.59 × 10^6^ km^2^. The current distribution area with high suitability covered ca. 4.87 × 10^5^ km^2^, with moderate suitability covered ca. 1.37 × 10^6^ km^2^, and the current habitats with low suitability covered ca. 1.73 × 10^6^ km^2^ (Table 2).

### 3.2. Important Environmental Variables

**Figure 3:**
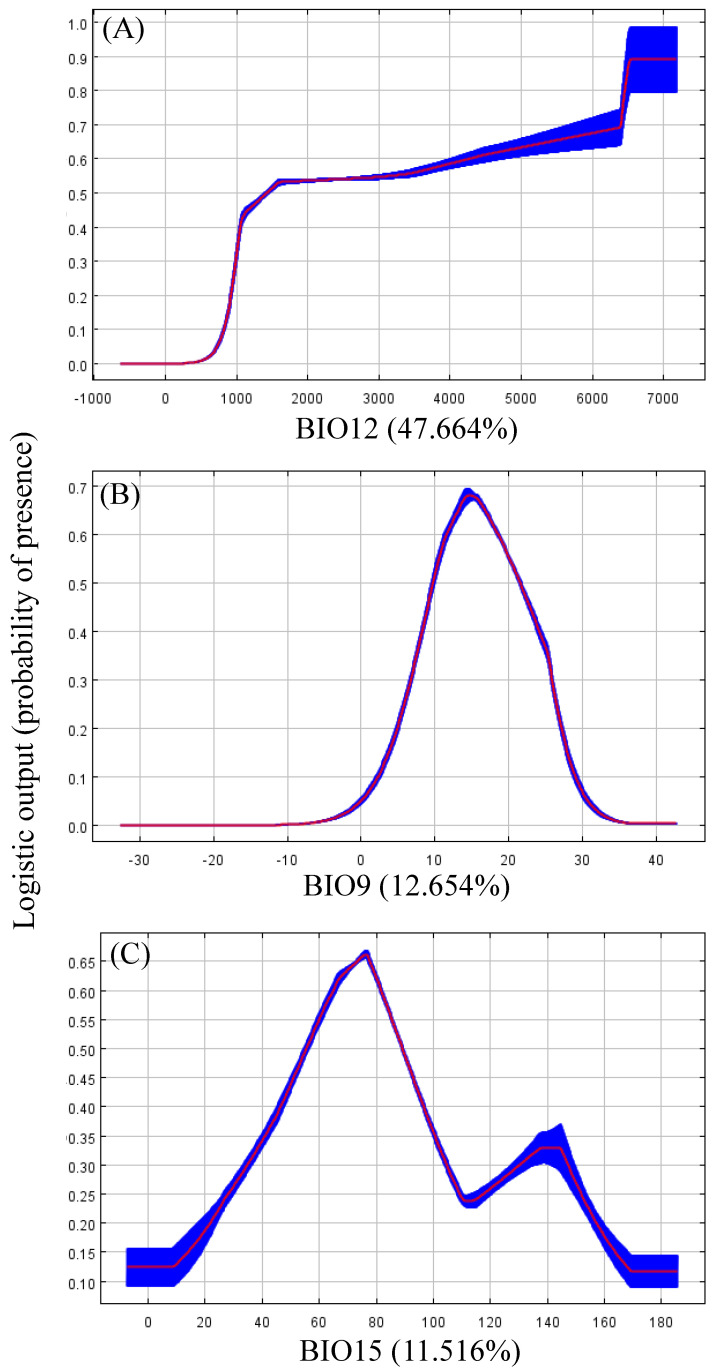
Response curves of three environmental variables greatly explain the spatial distribution of *N. chinensis*. (**A**), annual precipitation; (**B**), mean temperature of the driest quarter; (**C**), precipitation seasonality.

The Jackknife test showed that annual precipitation (BIO12, 47.664% of variation), mean temperature of the driest quarter (BIO9, 12.654% of variation), and precipitation seasonality (BIO15, 11.516%) well explained the distribution of *N. chinensis* (Table 3). Environmental factors contributing more than 10% were selected to be analyzed independently for their effects on the potential habitats. With annual precipitation between 1500 mm and 6300 mm, the suitability of *N. chinensis* ranges from 0.53 to 0.70, and with annual precipitation over 6300 mm, the suitability reaches a higher level (about 0.9) (Figure 3A). In high and moderate potential habitats of *N. chinensis*, the mean temperature of the driest quarter was between 9 °C and 23 °C, with the optimum temperature of the driest quarter being 14 °C (Figure 3B). In addition, there were two peaks for precipitation seasonality (coefficient of variation) (Figure 3C). When the coefficient of variation of seasonal precipitation was around 140, potential habitats presented low suitability values (0.2–0.4), but when the coefficient was around 78, it showed a high suitability of values (>0.6).

### 3.3. Habitat Selection

**Figure 4:**
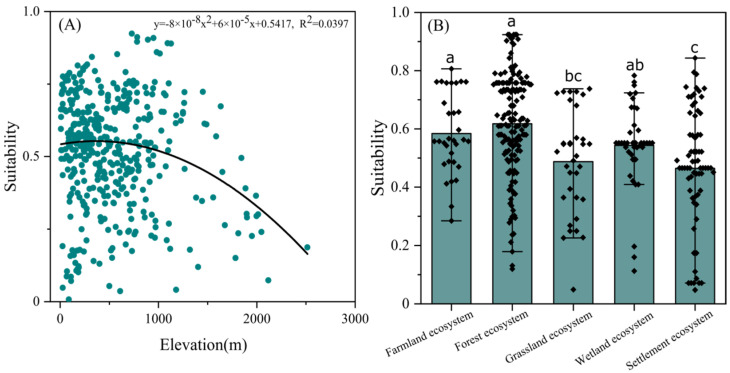
Effects of elevation (**A**) and ecosystem type (**B**) on habitat suitability of *N. chinensis*. Small letters indicated significant differences among five ecosystem types (farmland ecosystem, forest ecosystem, grassland ecosystem, wetland ecosystem, and settlement ecosystem) at *p* < 0.05 according to one-way ANOVA.

The elevation of 532 occurrences (8-2515 m a.s.l.) in *N. chinensis* was used to analyze the habitat bias. In those habitats over 1200 m a.s.l., both the number of habitats and their suitability values were gradually decreasing, while the higher suitability values mainly occurred between 500 and 1200 m a.s.l., suggesting a weak mid-domain effect for *N. chinensis* (*R*^2^ = 0.0397) (the maximum suitability values was around 0.9) (Figure 4A). When the ecosystem type was considered, potential habitats presented relatively high suitability values (>0.6) in forest ecosystems and moderate suitability values (0.4~0.6) in disturbed environments, such as farmland ecosystems, wetland ecosystems, and even settlement ecosystems (Figure 4B).

### 3.4. Future Changes in Suitable Habitat Area

**Figure 5:**
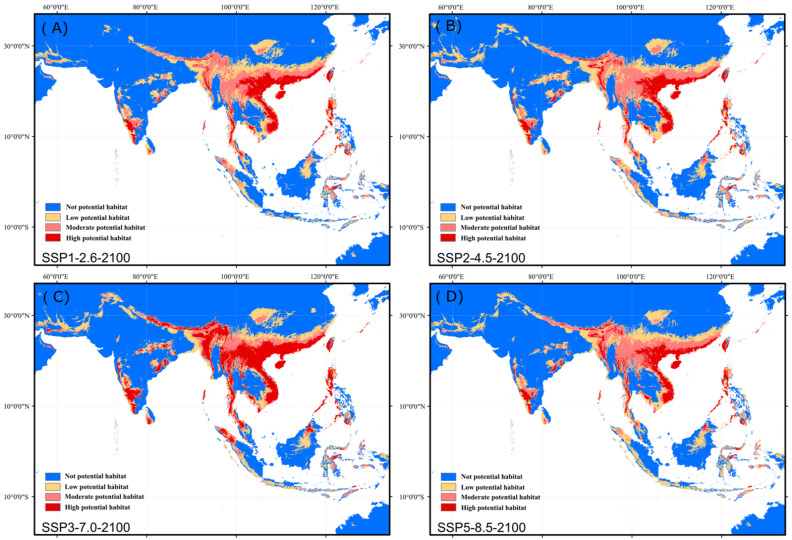
Prediction of the potential distribution area of *N. chinensis* in 2100 under four levels of greenhouse gas emission scenario: (**A**), the low-end SSP1-2.6 scenario; (**B**), the low-moderate SSP2-4.5 scenario; (**C**), medium-high SSP3-7.0 scenario; (**D**), the high SSP5-8.5 scenario.

Future climate scenarios (the 2100s) showed that the highly suitable habitats of *N. chinensis* were on the coast of south China, central and southern Vietnam, northern Laos, eastern Myanmar, Luzon Island of the Philippines, southwest India, Bangladesh, Bhutan, Nepal, and parts of the foothills of the Himalayas, and showed a tendency to expand to the north (Figure 5A–D). Future warming tends to extend high suitable habitats, including core distribution areas (Table 1). The modeling results showed that all habitat areas of *N. chinensis* under different levels of greenhouse gas emission scenario, amounting to ca. 4.20 × 10^6^ km^2^ (SSP1-2.6), ca. 4.43 × 10^6^ km^2^ (SSP2-4.5), ca. 4.62 × 10^6^ km^2^ (SSP3-7.0), and ca. 4.21 × 10^6^ km^2^ (SSP5-8.5), respectively (Table 2).

### 3.5. The Core Distribution Shifts

**Figure 6:**
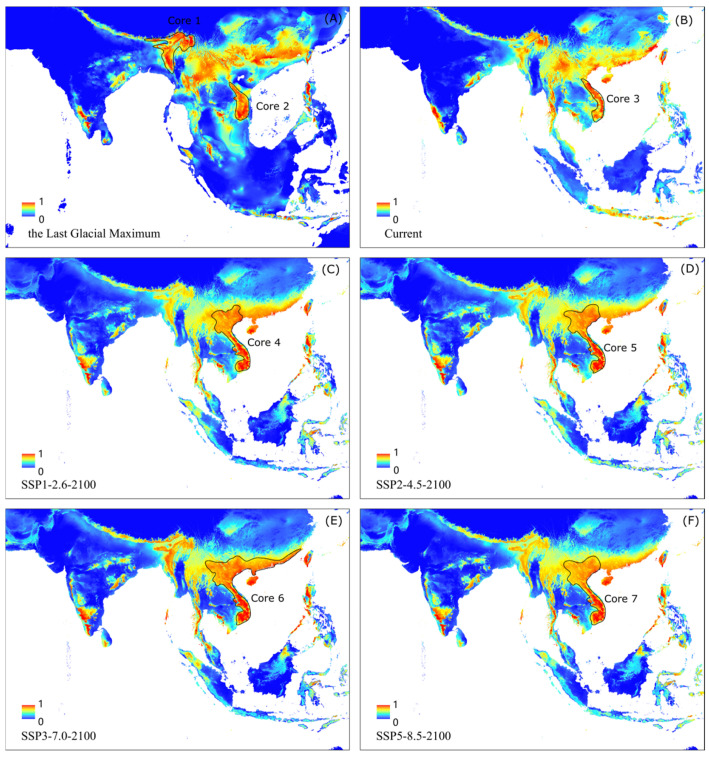
Prediction of core distribution areas under climate conditions: (**A**) historical climate; (**B**) current climate and future climate in 2100 under four scenarios, (**C**) SSP1-2.6, (**D**) SSP2-4.5, (**E**) SSP3-7.0, and (**F**) SSP5-8.5.

In the LGM, two core distributions were revealed: (i) the border area of northeast India, Bangladesh, Nepal, and Bhutan (Core 1), and (ii) south-central Vietnam (Core 2). The historical cores together cover ca. 2.37 × 10^4^ km^2^ (Figure 6A). One core distribution was detected under the current climate, which was mainly located in south-central Vietnam and covering ca. 1.67 × 10^4^ km^2^ (Figure 6B, Table 2). The core distribution area shifted north to the whole of Vietnam and even northward to more areas along the southeast coast of China under the four levels of greenhouse gas emission scenario (Figure 6C–F). Under the medium-high SSP3-7.0 scenario, the area of the core habitats of *N. chinensis* was maximized, about 6.22 × 10^5^ km^2^ (Table 2).

## 4. Discussion

All *Neurobasis* species are tropical that reside in hot and humid environments of eastern Asia [2]. Our results revealed that annual precipitation, mean temperature of the driest quarter, and precipitation seasonality contribute most to the distribution of *N. chinensis*. It prefers streams in forest ecosystems at mid-elevations (mainly distributed below 1200 m above sea level, preferring 500 to 1200 m), but it also tolerates habitats disturbed by human activities, such as farmland ecosystems and settlement ecosystems. In the current climate, the habitats of *N. chinensis* are located along a surface area of ca. 3.59 × 10^6^ km^2^. The core habitat area in historical climate was concentrated in two regions: (i) the border area of northeast India, Bangladesh, Nepal, and Bhutan (Core 1); (ii) south-central Vietnam (Core 2). However, the current core distribution remains only in south-central Vietnam. Under the four levels of carbon emission scenarios by 2100, the future distribution area of *N. chinensis* has a trend of active expansion. Under the low to moderate-high greenhouse gas emission scenario (SSP1-2.6 to SSP3-7.0 scenario), the trend showed an enhanced phenomenon, but under the high emission scenario, the expansion slowed down slightly. It seems that future warming will extend northward the suitable habitats of *Neurobasis* (including the core distribution area). The current southern habitats will be transformed into high potential suitable habitats under future warming scenarios.

### 4.1. Current and Future Potential Distribution

Northward (the Northern Hemisphere) and high-latitude expansion has been reported in many species of plants and insects [11,12]. Orr and Hämäläinen pointed out that Fujian, China, is the northernmost habitat of *N. chinensis* [2]; however, the modeling predicts that Shanghai, located in the north of Fujian, could also contain suitable habitats where species could occur under current climate conditions (Figure 2), which need to be confirmed by further fieldwork. The IUCN records that *N. chinensis* is widely distributed in South Asia (mainly the Indian peninsula) and Southeast Asia [3]. The occurrences of our collection and the predicted suitable habitats in South Asia are limited in the southwest (the southern foot of the Western Ghats) and northeast (the eastern Ghats near the Ganges Delta) of India. The southwest monsoon from the Indian Ocean makes landfall at the southern foot of the Western Ghats, while the northeast monsoon from the Asian continent bypass the Himalayas to reach the Ganges Delta, providing *N. chinensis* with favorable environment conditions (i.e., suitable temperature and abundant precipitation) [4]. In addition, the Thar Desert in the northwest, the Himalayas in the north, and the Deccan Plateau in the central region block the monsoons, indicating that the species is only suitable for the foothills of the southwest of the Indian peninsula and the Ganges Delta in the northeast of South Asia.

Future warming is inevitable, and indigenous geographical properties are changing, which may affect the natural distribution of many species [6,27,28,29,30,31]. In the present study, we predicted the potential habitats will expand under four levels of greenhouse gas emissions scenario by 2100. The high potential habitats for this tropical species moved northward under four scenarios. Moreover, in Nepal, Bhutan, and East India, high potential habitats expanded to high elevations as greenhouse gas emissions increased. Under the medium-high SSP3-7.0 scenario, the high potential habitats will expand to the largest area, and the total potential distribution for *N. chinensis* in the future will reach the maximum. However, under the high emission scenario (SSP5-8.5), the expansion slowed. These indicated that under the future warming, habitat loss for *N. chinensis* will not occur and even help them colonize north or higher elevation habitats. However, moving north or to high-elevation habitats is not necessarily a good thing for species native to northern or high altitudes and may lead to a series of potential problems that threaten the stability of northern ecosystems. Overall, the *N. chinensis* does not appear to be under major threat for the next century, but *N. anderssoni*, a rare Chinese endemic species of the genus *Neurobasis*, has not been seen for decades, which deserves our attention.

### 4.2. Habitat Selection and Critical Climate Factors

Our study detected a weak mid-domain effect (i.e., favoring mid-elevation) and had relatively high suitability in forest ecosystems. Some researchers suggested that *N. chinensis* inhabited woodland streams at 500–1200 m a.s.l. [4], while others believed that it lived in mountainous areas at 1300–2000 m a.s.l. [2]. Empirically, it is distributed in a wider range (0–2500 m a.s.l.) of elevation in the Oriental region. In contrast, our studies showed that the distribution with high suitability was at the low and mid-altitude forest below 1200 m a.s.l., with a preference for habitats between 500 and 1200 m a.s.l. Related studies also stated that *N. chinensis* prefers forest and woodland streams with grassy borders but also breeds in disturbed environments [2] (i.e., farmland and settlement ecosystems counted in this study). Some authors hold that it flies throughout the year in much of its range [1] but seasonally in the north and at higher elevations [2], which may be related to the combined hydrothermal conditions in the north or at higher elevations.

Damselflies cannot access resources, grow, and reproduce without water bodies. They usually lay their eggs in forest streams, wetlands, or shallow lakes and spend their larva stage underwater. *N. chinensis* perches in the mountains of the tropics, and the female lays eggs on submerged decaying logs in streams during the southwest monsoon [4]. Tropical forest streams are fed by precipitation; therefore, variation in precipitation is probably the main factor determining the potential habitats. We confirmed this conjecture in our model analysis, and the Jackknife test showed that annual precipitation and precipitation seasonality (the contribution together account for about 60%) were crucial factors in the distribution of *N. chinensis*. The temperature during the dry season (or winter) in the tropics is often an important driver of their life history (i.e., early larva of dragonflies) [32]. We also detected a high explanation of temperature in the driest quarter as an important factor for the potential distribution of *N. chinensis*. Published research demonstrated a link between temperature changes and insect outbreaks using 51 years of empirical data on the tea tortrix *Adoxophyes honmai* (Lepidoptera: Tortricidae) [33]. They suggested that temperature change is associated with changes in ecosystem stability [6,33]. The effect of future temperature change on the system stability in tropical regions is lower than that in temperate regions. Although *N. chinensis* was not predicted to be threatened by global warming, their tendency to invade northward or to higher altitudes could be detrimental to the survival of native northern or mountaintop species.

### 4.3. Historical Core Distribution and Its Future Shift

**Figure 7:**
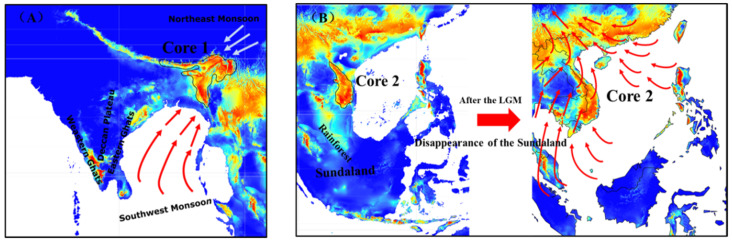
The causes of historical core habitats of *N. chinensis* in South and Southeast Asia at the last glacial maximum. (**A**), Core 1 is affected by the monsoons; (**B**), Core 2 is affected by monsoons and the disappearance of Sundaland.

It is highly possible, yet not all of the time, that the historical core distribution was also a refugium for species with higher diversity and a focus on conservation biology [34]. Nevertheless, understanding its location and shift has important implications for the dispersal of populations and long-term conservation [34]. In this study, we revealed two historical cores of *N. chinensis* and predicted how it will shift in a warming future. Between the two cores, historical Core 1 was in the inland region of the Bay of Bengal (the border area of northeast India, Bangladesh, Nepal, and Bhutan), and Core 2 was in south-central Vietnam on the coast of the Beibu Gulf, and they may be associated with the Indian Ocean monsoon (southwest monsoon), northeast monsoon (Figure 7A) and the Sundaland disappearance after the LGM (Figure 7B).

The emergence of Core 1 seems to coincide with two monsoon paths and its climate history in the Bay of Bengal region. Ding and Fang (2006) revealed that at the LGM, the northeastern monsoon was strong in intensity with the active upwelling, then cut off by the Deccan Plateau and the Eastern Ghats, producing rainfall in the north of Bay of Bengal in the oxygen isotope stage 2 (MIS 2) [35]. The southwestern monsoon from the northern Indian Ocean was strong in early and late oxygen isotope stage 3 (MIS 3), and monsoonal rains increased in the north of the Bay of Bengal [35]. These changes may be associated with our identified Core 1 in the inland region of the Bay of Bengal (Figure 7A).

However, only one core distribution was identified under the current climate, i.e., the Core 2, which may be linked to the disappearance of the Sundaland in Southeast Asia during the LGM (Figure 7B). Based on the prediction of the future climate warming under the four shared socioeconomic pathway (SSP) greenhouse gas concentrations, the historical Core 2 of *N. chinensis* will expand from south-central Vietnam to northern Vietnam and even southern China (Figure 6). Recent marine palynological evidence indicated that at the LGM, Sundaland was covered with rainforests [36,37]. Rainforests tend to have high biodiversity and provide a refugium for many species. For example, studies speculated that rainforest refugia for termites and murine rodents may exist on the Sundaland [38,39]. Using the stable carbon isotope composition of ancient cave guano profiles, researchers found that a substantial forest contraction occurred during the LGM in both peninsular Malaysia and Palawan [40], while rainforest was maintained in northern Borneo. After the LGM, however, the Sunda continental shelf was gradually submerged. Therefore, the edge of Sundaland, south-central Vietnam, retains a high level of diversity that might be associated with our prediction of core distribution (Core 2). This further supports south-central Vietnam (the north of the Sundaland) as a historical core distribution of *N. chinensis*. Moreover, much of Vietnam is affected by the East Asian monsoon. From the last glacial maximum to the mid-Holocene, the East Asian monsoon rain belt migrated to the northwest due to warming [41]. Typically, the East Asian summer monsoon plays a crucial role in interhemispheric heat and moisture transport and serves as the main moisture supply for East Asia [42]. The historical core distribution of *N. chinensis* seems to benefit from the East Asian monsoon, and the core habitats after the last glacial maximum may be linked to the warming-induced northwestward migration of the East Asian monsoon rain belt [41].

## 5. Conclusions

Currently, *N. chinensis* mainly inhabits forest ecosystems below 1200 m a.s.l. prefer habitats between 500 and 1200 m a.s.l., and has a potential distribution of ca. 3.59 × 10^6^ km^2^. There were two core distributions at the LGM, but only one remains currently and was concentrated in south-central Vietnam. The potential distribution was predicted to increase in the warming future and have the largest area under medium-high scenario SSP3-7.0. The core distribution area, high suitability habitats, and even the whole species’ range will shift northward. Our studies provide useful information for the conservation and management of *N. chinensis.*

## Figures and Tables

**Table 1:** Environmental variables and elevation information that may shape species distribution.

Abbreviations	Environmental Variables
BIO1	Annual mean temperature
BIO2	Mean diurnal range (mean of monthly max temperature–min temperature)
BIO3	Isothermality ((BIO2/BIO7) × 100)
BIO4	Temperature seasonality (standard deviation × 100)
BIO5	Max temperature of warmest month
BIO6	Min temperature of coldest month
BIO7	Temperature annual range (BIO5–BIO6)
BIO8	Mean temperature of wettest quarter
BIO9	Mean temperature of driest quarter
BIO10	Mean temperature of warmest quarter
BIO11	Mean temperature of coldest quarter
BIO12	Annual precipitation
BIO13	Precipitation of wettest month
BIO14	Precipitation of driest month
BIO15	Precipitation seasonality (coefficient of variation)
BIO16	Precipitation of wettest quarter
BIO17	Precipitation of driest quarter
BIO18	Precipitation of warmest quarter
BIO19	Precipitation of coldest quarter
Ele.	Elevation (meter a.s.l.)

**Table 2:** Change of suitable habitat area and core distribution (km^2^) of *N. chinensis* in current and future under four levels of greenhouse gas emission scenario.

Scenario	High Suitable Habitat	Moderate Suitable Habitat	Low Suitable Habitat	Core Habitat	Total
Current	4.87 × 10^5^	1.37 × 10^6^	1.73 × 10^6^	1.67 × 10^4^	3.59 × 10^6^
SSP1-2.6	7.30 × 10^5^	1.32 × 10^6^	2.16 × 10^6^	5.34 × 10^4^	4.20 × 10^6^
SSP2-4.5	7.15 × 10^5^	1.45 × 10^6^	2.27 × 10^6^	5.70 × 10^4^	4.43 × 10^6^
SSP3-7.0	1.52 × 10^6^	1.08 × 10^6^	2.02 × 10^6^	6.22 × 10^5^	4.62 × 10^6^
SSP5-8.5	7.37 × 10^5^	1.35 × 10^6^	2.12 × 10^6^	4.22 × 10^4^	4.21 × 10^6^

**Table 3:** Percentage explanation and permutation importance of the bioclimatic variables.

Abbreviation	Variable	Explanation	Importance
BIO2	Mean diurnal range in temperature	2.237%	0.445
BIO5	Max temperature of warmest month	8.500%	7.356
BIO8	Mean temperature of wettest quarter	2.195%	4.198
BIO9	Mean temperature of driest quarter	12.654%	32.538
BIO12	Annual precipitation	47.664%	10.636
BIO14	Precipitation of driest month	0.515%	1.090
BIO15	Precipitation seasonality	11.516%	8.095
BIO18	Precipitation of warmest quarter	5.036%	19.720
BIO19	Precipitation of coldest quarter	9.683%	15.921

## Data Availability

The data presented in this study are available on request from the corresponding author.
